# Acoustic Mapping of Thermohaline Staircases in the Arctic Ocean

**DOI:** 10.1038/s41598-017-15486-3

**Published:** 2017-11-09

**Authors:** Christian Stranne, Larry Mayer, Thomas C. Weber, Barry R. Ruddick, Martin Jakobsson, Kevin Jerram, Elizabeth Weidner, Johan Nilsson, Katarina Gårdfeldt

**Affiliations:** 1 Department of Geological Sciences, Stockholm University, Stockholm, Sweden; 2 Center for Coastal and Ocean Mapping, University of New Hampshire, Durham, New Hampshire USA; 3 Bolin Center for Climate Research, Stockholm University, Stockholm, Sweden; 4 Department of Oceanography, Dalhousie University, Halifax, Nova Scotia Canada; 5 Department of Meteorology, Stockholm University, Stockholm, Sweden; 6 Department of Chemistry and Chemical Engineering, Chalmers University of Technology, Göteborg, Sweden

## Abstract

Although there is enough heat contained in inflowing warm Atlantic Ocean water to melt all Arctic sea ice within a few years, a cold halocline limits upward heat transport from the Atlantic water. The amount of heat that penetrates the halocline to reach the sea ice is not well known, but vertical heat transport through the halocline layer can significantly increase in the presence of double diffusive convection. Such convection can occur when salinity and temperature gradients share the same sign, often resulting in the formation of thermohaline staircases. Staircase structures in the Arctic Ocean have been previously identified and the associated double diffusive convection has been suggested to influence the Arctic Ocean in general and the fate of the Arctic sea ice cover in particular. A central challenge to understanding the role of double diffusive convection in vertical heat transport is one of observation. Here, we use broadband echo sounders to characterize Arctic thermohaline staircases at their full vertical and horizontal resolution over large spatial areas (100 s of kms). In doing so, we offer new insight into the mechanism of thermohaline staircase evolution and scale, and hence fluxes, with implications for understanding ocean mixing processes and ocean-sea ice interactions.

## Introduction

There is enough heat contained in intruding Atlantic Ocean waters to melt all Arctic sea ice within a few years^1^. Mixing of the warm Atlantic water into colder Arctic surface water and sea ice is, however, limited by a cold halocline^2–4^. How much heat penetrates upward through the halocline and reaches the sea ice is debatable^5^, but vertical heat transport through the halocline layer can significantly increase in the presence of double diffusive convection^6^. Such convection can occur when salinity and temperature gradients share the same sign, and often leads to the formation of thermohaline staircases^7^. Since the presence of thermohaline staircases facilitates larger heat flow through the halocline (ref.^1^ assumes a factor of 40 larger), there is an impact of these formations on the Arctic Ocean in general^8^ and the Arctic sea ice cover in particular^1^.

**Figure 1:**
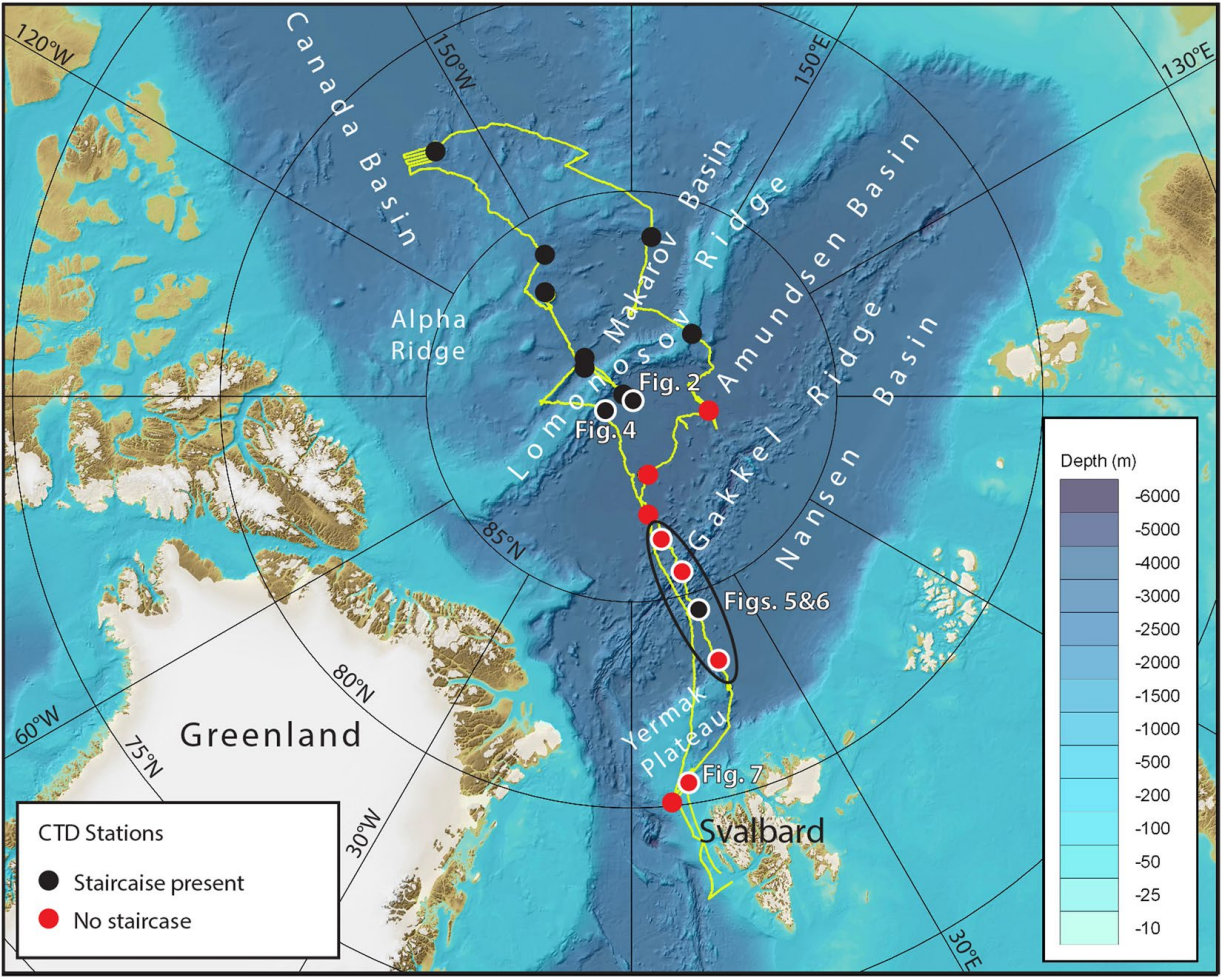
Overview of the *Oden* ship track (yellow line) during the Arctic Ocean 2016 expedition, between 8 August and 19 September, 2016, departing from and returning to Svalbard. Broadband water column backscatter data were collected continuously along the ~8700 km vessel track outside the territorial waters of Svalbard. Conductivity-temperature-depth (CTD) stations are shown as black dots and red dots, depending on presence of thermohaline staircases. White rings around dots indicate CTD stations shown in figures. . Background bathymetry is IBCAO v3 in Universal Polar Stereographic projection with the latitude of true scale at 75° N. The map was made with QGIS software, version 2.18.11 (www.qgis.org).

The manner in which thermohaline staircases are formed and their relative contribution to heat flow is, however, not well known^9,10^. A central challenge to understanding the role of double diffusive convection in vertical heat transport is one of observation. Conductivity, temperature and depth (CTD) profiles have traditionally been used to provide detailed pictures of vertical water column structure with high resolution, but only at discrete locations^8,10–13^. Acoustic observations have been used to observe the physical properties and structure of the ocean. Seismic systems, for example, have been used to synoptically map oceanographic features^14^, although these measurements lack the vertical resolution to resolve ocean structure at the scale of Arctic thermohaline staircases. Measurements with higher-frequency acoustic echo sounders have been used to relate acoustic scattering observations to variations in sound speed and density associated with turbulent microstructure^15–17^. It has even been suggested that thermohaline staircases could be observed using acoustic echo sounders^18^. Here, we unequivocally demonstrate that thermohaline staircases (and by extension other similarly sharp gradients in ocean temperature and salinity) can be acoustically mapped in the open ocean and use these observations to gain initial insights into the spatial and temporal evolution of the staircases. Our observations were collected with a commercially available Simrad EK80 broadband (15–25 kHz) echo sounder deployed on the icebreaker *Oden* in the ice-covered waters of the high Arctic Ocean during the summer of 2016 (Fig. 1).

## Methods

### Broadband water column acoustic data collection

The broadband water column backscatter data presented here were collected with a Simrad EK80 split-beam scientific echosounder (SBES). This system was operated continuously aboard the Swedish icebreaker *Oden* over a vessel track of approximately 8700 km during the Arctic Ocean 2016 expedition. The expedition departed Svalbard on 8 August and returned to Svalbard on 19 September, 2016.

The SBES consisted of a Simrad EK80 broadband transceiver transmitting through a standard Simrad ES18-11 transducer, protected by an ice window behind an ‘ice knife’ near the bow of the vessel. This transducer model is widely installed among fishery research vessels, typically operating at 18 kHz with a −3 dB beamwidth of 11°. In 2014, the transducer model was tested with a Simrad EK80 broadband transceiver and determined to have a useable two-way frequency response over 15–25 kHz that was suitable for open ocean data collection. Thus, a frequency range of 15–25 kHz was used throughout the EK80 data collection period on *Oden*.

Transmit power was maintained at the maximum setting of 2000 W to offset losses through the ice protection window and improve signal-to-noise (SNR) characteristics, especially during noisy hull-ice interactions. Transmission pulse lengths were adjusted over a range of 1–8 ms, in an effort to minimize the extent of autocorrelation sidelobes (better with shorter pulses) while maximizing the SNR (better with longer pulses). All EK80 operation was controlled and monitored around-the-clock using the Simrad user interface to adjust pulse length and range recording duration. Data were logged directly to large storage arrays in the Simrad raw format.

Position and attitude information were provided to the sonar as an integrated solution by a Seapath Seatex 330 vessel GPS and motion reference system. Vessel motion was minimal (typically less than 1° pitch and roll, in the data presented here) and thus does not appreciably affect the observations of horizontally-oriented backscattering layers occupying broad portions of the beam.

The EK80 was synchronized with the on-board multibeam echosounder (12 kHz) and subbottom profiler (2.5–7 kHz) throughout the data collection period. A small delay was applied to the EK80 transmit-receive cycle trigger in order to avoid transmission interference from the other systems in the earliest portion of the EK80 receive cycle, corresponding to the upper water column region of interest. The *Oden* worked in tandem with Canadian icebreaker *Louis S*. *St*. *Laurent* (*LSSL*) during the first half of the expedition; due to the separation of the vessels, frequency ranges, and beam patterns, the *LSSL*’s mapping systems were not observed to interfere with the EK80 aboard *Oden*.

### EK80 post processing methodology

The dataset collected with the EK80 was match filtered with an ideal replica signal using a MATLAB software package provided by the system manufacturer, Kongsberg Maritime (Lars Anderson, personal communication). After match filtering, ship-related noise was found within the signal band, and so a bandpass filter (16 and 22 kHz cut-off frequencies) was applied to the data to remove it. Ranges from the transducer were calculated using the cumulative travel times through sound speed profile layers based on the nearest (in time) CTD profile. These ranges were then converted to depths by compensating for the transducer location relative to waterline on *Oden* and heave of the vessel.

### EK80 extended target calibration procedure

The EK80 was calibrated onboard the *Oden* on 1 September 2015, following a standard methodology described by^19^. A 64 mm copper sphere of known acoustic properties was suspended on a monofilament line and moved through the SBES field of view. The calibration data were collected in relatively calm seas and atmospheric conditions while the *Oden* drifted. All propulsion systems were secured during the calibration procedure in order to reduce noise in the water column. A CTD was collected immediately before calibration operations.

Utilizing a target strength sphere model based on the work by^20^ and^21^ (MATLAB software package available at www.ices.dk), a calibration offset (*C* = 8.5 dB, averaged over the transducer beam width) was calculated using a temperature of 0 °C and a salinity of 34.5 at the sphere depth of approximately 80 m. This calibration offset represents the difference between the nominal target strength (*TS*) observed by the EK80, as predicted after match filtering, and the true TS of the sphere. The offset is then used in subsequent measurements of *TS*.

### Estimates of the reflection coefficient from EK80 observations

The *TS* of an ideally smooth layer is a function of both the reflection coefficient (*R)*, and the ensonified area (*A*). Here, we assume that *A* is limited by the width of the EK80 beam (rather than the length of the pulse), such that A can be estimated as

$$A(z)=\pi {(\tan (\phi )z)}^{2},$$

where φ is half the beam width and *z* is the depth in the sonar reference frame. Following^22^ the *TS* for a layer at depth *z*, with reflection coefficient *R*, can then be estimated as

$$TS(z)=20lo{g}_{10}R+10{lo}{{g}}_{10}(A(z)).$$

For our estimates of observed *R*, we simply invert the above equation to solve for *R*:

$$R={10}^{(TS-10lo{g}_{10}A)/20},$$

where *TS* is the calibrated observation from the EK80. This simple approach does not account for a variety of phenomena that would provide further insight into the nature of the layer (e.g., layer roughness or a more refined stochastic treatment of the data), but serves the purpose of this work by allowing us to make estimates of *R* that help identify the physical properties of the water as the cause of the reflection.

### CTD

CTD data were collected with a SeaBird 911 equipped with dual SeaBird temperature (SBE 3) and conductivity (SBE 04 C) sensors. The CTD data files were post processed with SBE Data Processing software, version 7.26.6 (available at www.seabird.com). The alignment parameter was tuned following the suggested method described in the SBE Data Processing manual (available at www.seabird.com). All CTD data presented are binned in 10 cm vertical averages.

The reflection coefficient from CTD data (*R*_*CTD*_) was calculated through

$${R}_{CTD}(i)=\frac{\eta (i)-\eta (i-1)}{\eta (i)+\eta (i-1)},$$

where each element *i* has a corresponding depth *z*(*i*), and the depth of ${R}_{CTD}(i)$ is the average of *z*(*i*−1) and *z*(*i*), and η is the impedance given by

$${\rm{\eta }}(z)=V(z)\rho (z),$$

where *V* is the sound speed and *ρ* the seawater density. The accuracies of the pressure, conductivity and temperature sensors are 0.0015%, 0.0003 S/m and 0.001 °C, respectively (www.seabird.com). All conversions (salinity, density and sound speed) were made according to the International Thermodynamic Equation of Seawater^23^.

## Results

**Figure 2:**
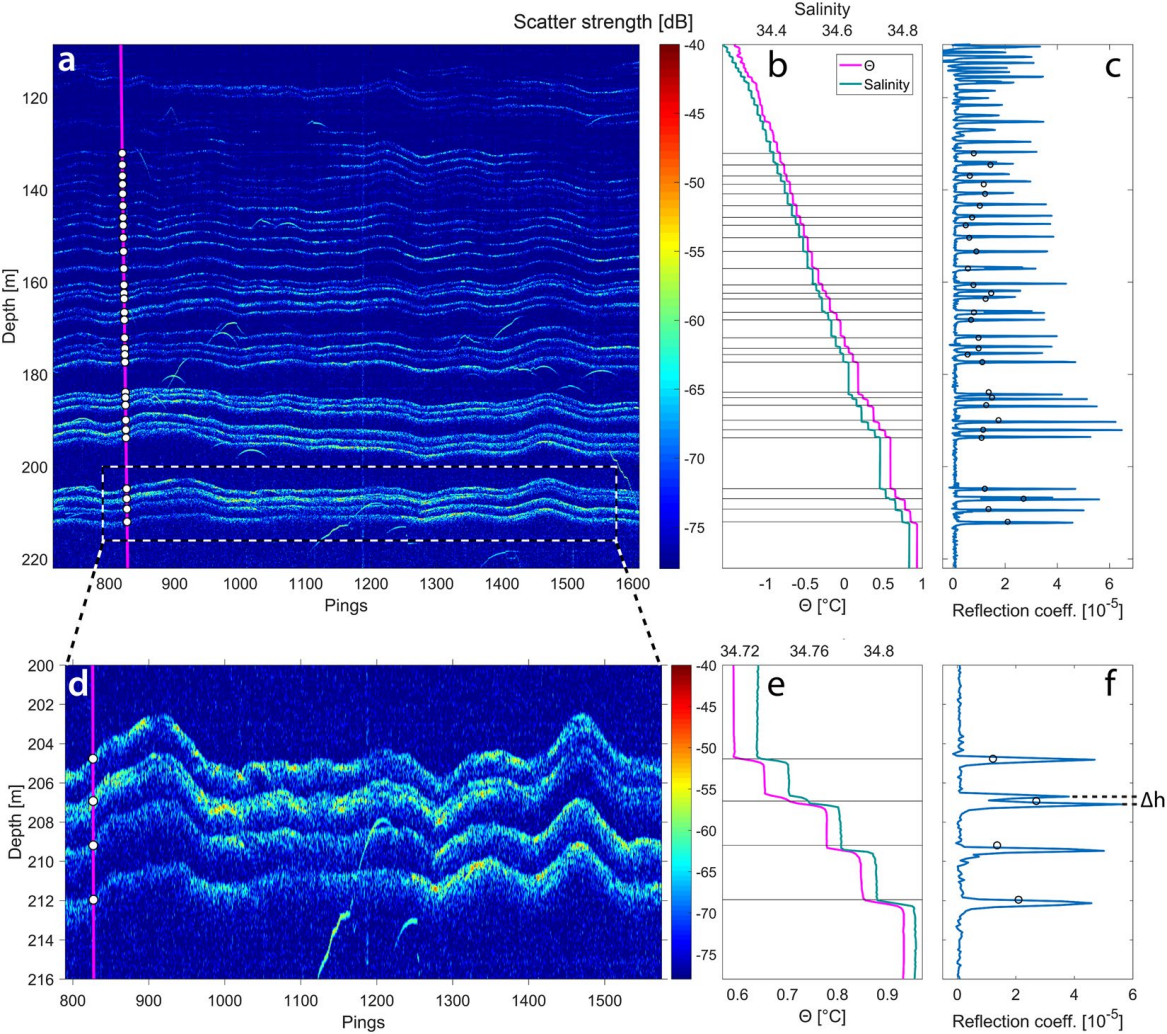
Acoustic observations of a thermohaline staircase. (**a**) Processed EK80 echogram with 8 ms pulse length covering 2.5 h and a distance of 7 km, with CTD cast (magenta line) and layer depths derived from the echogram scatter strength (white circles). (**b**) CTD potential temperature with reference at the surface (Θ) and salinity profiles with black horizontal lines indicating the depth of the individual layers identified in the echogram (white circles in **a**). (**c**) Reflection coefficient derived from CTD salinity and temperature profiles (blue line) and reflection coefficients estimated from the calibrated target strength in each layer (black circles, see Supplementary Information for details) at depths derived from the echogram (white circles in **a**). (**d–f**) Same as (**a–c**) but over the narrower depth range indicated in the dashed box in **a**. ∆h (=0.4 m) in (**f**) is the distance between two reflection coefficient peaks, partly visible in (**d**) and represents the minimum spacing visually separable between acoustic horizons (observed vertical resolution). Echoes from fish are seen throughout the data (**a**,**d**) as irregular, sometimes hyperbolic, traces.

The EK80 data were processed to produce high-resolution acoustic reflectivity images. The reflectivity images show a series of closely-spaced weak scattering layers (Fig. 2a,d) that correspond, one-for-one, with small discrete changes in temperature (~0.05 °C) and salinity (~0.015) recorded in co-located CTD casts (Fig. 2b,e). Instances of thin layer interference, as observed in tank experiments^17^, were not seen in these field observations. The acoustic reflection coefficients of these thermohaline steps, calculated from the CTD profiles are approximately four orders of magnitude smaller than those typical of seafloor reflections, and are in close agreement with the reflection coefficients determined from the acoustic target strength of the reflections (Fig. 2c,f). This reinforces the view that the layers seen in our acoustic data are the direct result of the impedance contrasts (i.e., gradients in the product of sound speed and density) at the layer interfaces. The reflection coefficients derived from the acoustic data are consistently slightly lower than the theoretical values (calculated from the CTD profiles), possibly due to deviations in the layer from a smooth surface, incoherent scattering due to turbulent microstructure associated with the layer, or imperfections in the assumptions used in converting the raw acoustic data to a reflection coefficient. It is also possible that target strength variability might be related to intermittent turbulent microstructure at the layer interfaces^17^.

Thermohaline staircases were seen in temperature and salinity profiles at 14 of the 24 CTD casts made during the expedition (Fig. 1). No staircase extended deeper than 300 meters. This depth range is consistent with those reported in the literature (140–350 m^8,10,12,13^) and our acoustic measurements captured their full depth extent. With one exception (in the Nansen Basin), staircases did not occur in the Eurasian Basin south of 89°N, while they were consistently observed in the Amerasian Basin (Fig. 1).

The vertical separations of individual steps in Arctic staircases determined from CTDs are typically between 0.5 and 5 m^8,10,12,13^. The broad bandwidth of the EK80, as configured for this work, provides a theoretical range resolution of about 0.10 m, which allows for the visual identification of individual steps within the thermohaline staircases separated by as little as 0.4 m (Fig. 2d–f). The vertical resolution of the EK80 is higher than that achieved with towed multi-channel seismic systems using 10–300 Hz sources^14,24,25^ by a factor of 20. Thus, broadband echo sounders offer the opportunity to image staircases at full resolution over large spatial scales.

The sampling (ping) rate of the EK80 echo sounder was typically around one ping per 10 s which is significantly higher than other means of observing Arctic staircases. Ice tethered profiler stations have a sampling rate of approximately every 8 h^26^, and CTD ‘tow-yoing’ from ice stations and icebreakers approximately every 10 minutes^10,12^. We have thus been able to observe, for the first time, the continuous high-frequency vertical displacements of individual steps within an Arctic thermohaline staircase caused by internal waves. By analyzing a representative portion of our data, we find a root-mean-square vertical displacement of individual steps to be around 2.5 m which is on the same order of magnitude as reported previously^12^.

**Figure 3:**
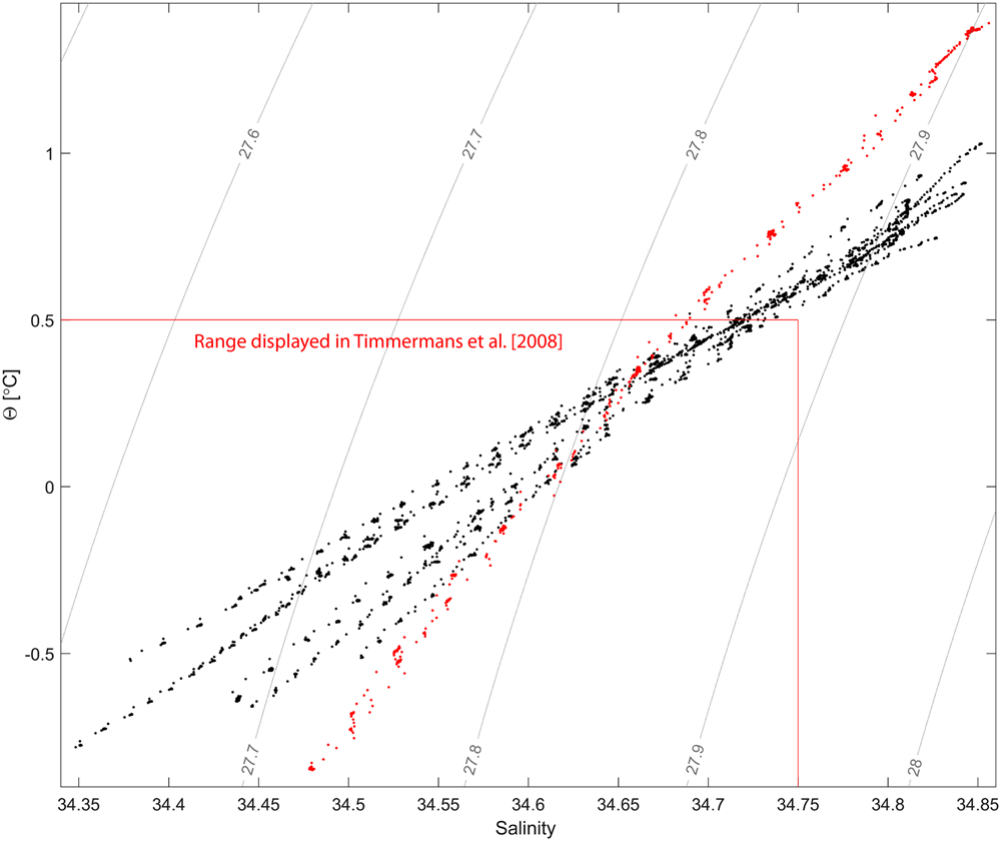
Salinity and potential temperature over the range of the individual staircases found in CTD data (green dots in Fig. 1), with contours showing potential density anomaly (with reference to the surface). Red scatter are from the isolated staircase (black and magenta profiles in Fig. 5).

**Figure 4:**
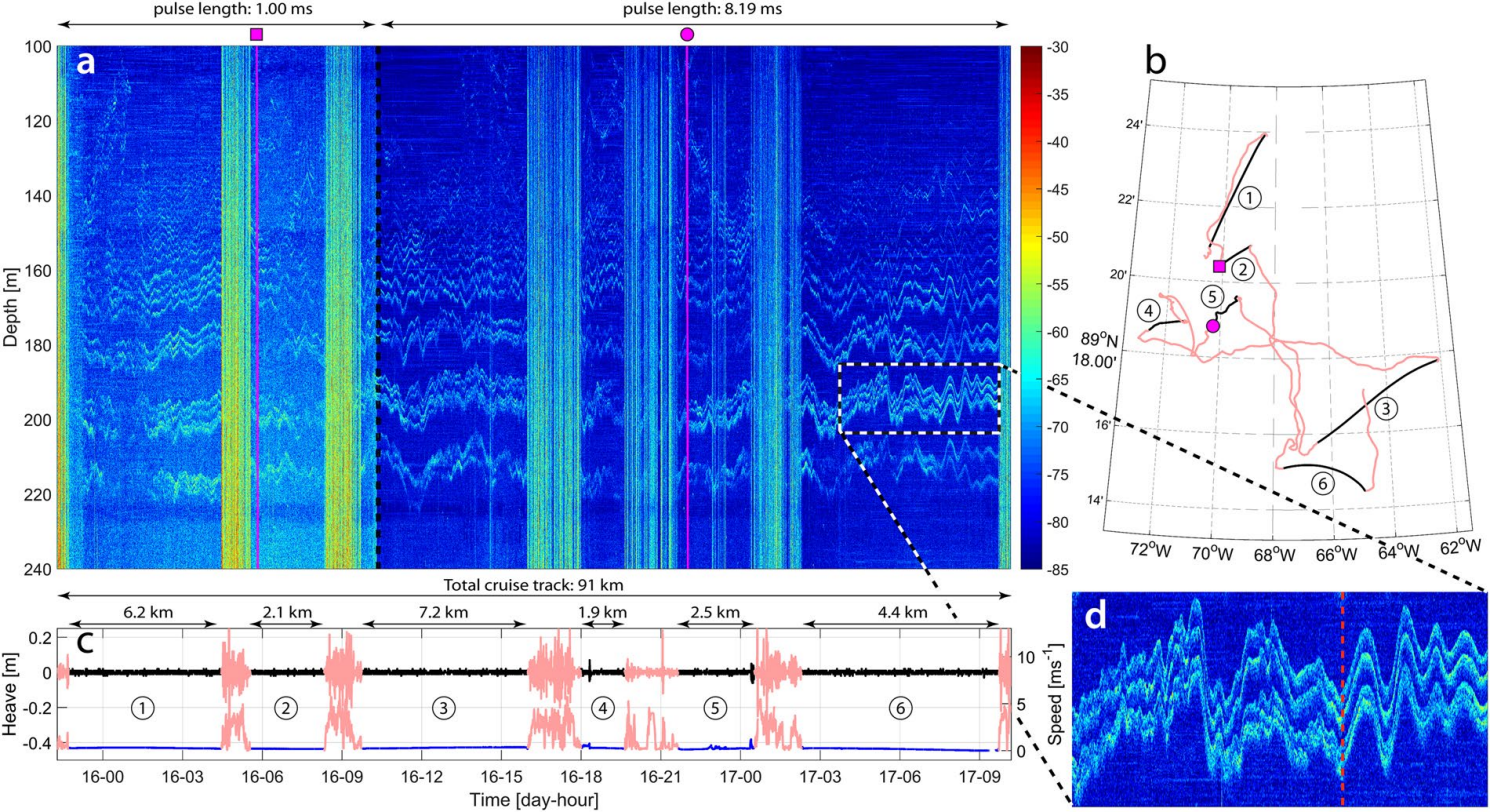
Continuous tracking of an Arctic thermohaline staircase over 36 hours. (**a**) EK80 echogram with color-bar indicating the scattering strength (dB). (**b**) Cruise track (pink) over a total of 91 km with drift stations (black numbered tracks) and two CTD stations with corresponding symbols shown on top of panel **a**. (**c**) Heave (black) and speed over ground (blue) over drift stations (corresponding to numbers in **b**) and while breaking ice (pink). (**d**) Enlargement of the dashed box in (**a**) showing evidence of shear instabilities with a transition (dashed red line) from unstable to stable conditions. Note that little or no useful acoustic data, in terms of ocean stratification, are obtained while breaking ice.

**Figure 5:**
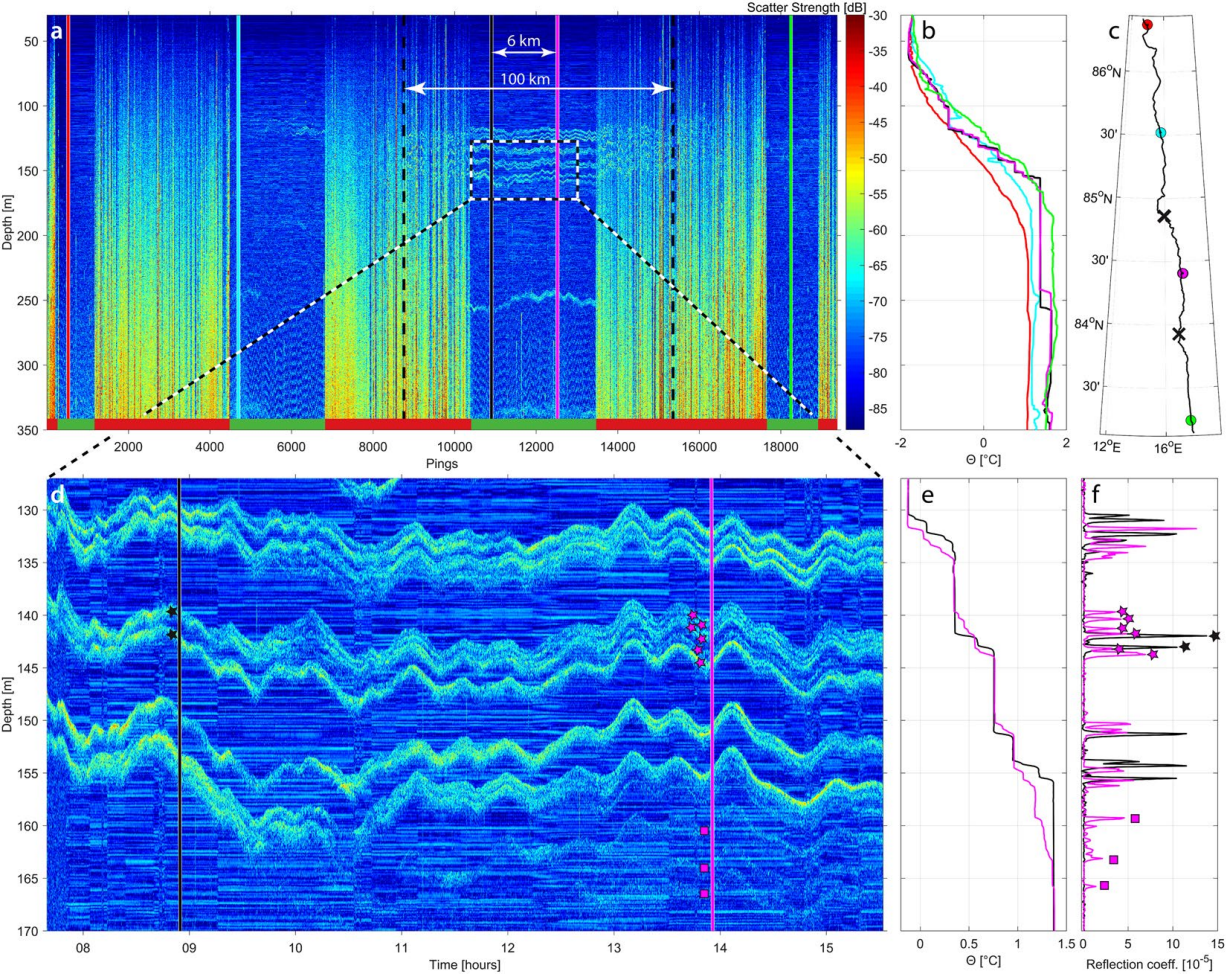
Isolated Arctic staircase. (**a**) EK80 echogram (2 ms pulse length) with red/green horizontal bars on bottom indicating periods of ice breaking (red) and drifting (green) and solid vertical lines indicating CTD casts. Also shown are the staircase edges (vertical dashed lines) indicating a lateral extension of the staircase of about 100 km of a 433 km vessel track segment spanning 64 h. (**b**) CTD temperature profiles. (**c**) Vessel track with locations of the CTD casts (circles, note that the black circle is masked by the magenta circle) and the staircase edges (black crosses). (**d**) Magnification of the dashed box in (**a**) with corresponding CTD potential temperature profiles (**e**) and reflection coefficients (**f**). Stars in (**d** and **f**) indicate how two steps (black) separate into six (magenta), and magenta squares in (**d** and **f**) indicate new layers forming in between the two stations.

By plotting potential temperature versus salinity of individual layers, sampled from ice-tethered profilers, *Timmermans et al*. identified a coherent thermohaline staircase covering essentially the whole Canada Basin (~1 × 10^5^ m)^13^. *Padman and Dillon* determined the staircase coherency by tracking the vertical position of individual layers in a sequence of temperature profiles^27^. They found that this method required less than 15 m separation between neighboring profiles and were only able to trace individual layers laterally for up to 600 m. *Sirevaag & Fer* applied both methods to a collection of about 500 profiles from the central Arctic Ocean (with average distance between profiles of 300–400 m) but found no significant lateral coherency^10^. Although there is some banding tendency in the potential temperature versus salinity plot (Fig. 3), we do not observe the lateral coherency reported in reference^13^ in the presented data. However, we were able to track a staircase continuously (with some interruptions due to ice breaking in between drift stations) for 36 h over a ship track of 91 km (Fig. 4a), and identify an isolated staircase, extending about 100 km laterally, in the Nansen Basin (Fig. 5). There is no reason to expect a limit for how far or for how long a staircase can be tracked acoustically as long as the impedance contrast within the steps is high enough to be detected. The apparent minimum detectable reflection coefficient in these data, derived from visually comparing the acoustic data with ocean stratification data, is ~1 × 10^−5^.

**Figure 6:**
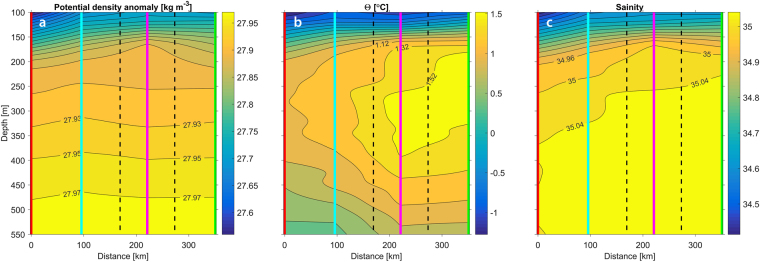
Anticyclonic intrathermocline eddy. Smoothed fields (profiles filtered with a low-pass Gaussian filter with ~0.1 m^−1^ cut-off frequency) of (**a**) potential density anomaly (with reference to the surface), (**b**) potential temperature and **c** salinity, with vertical colored lines corresponding to CTD profiles and dashed black lines to the staircase edges shown in Fig. 5.

No staircases were seen in the Eurasian Basin south of 89°N except for one in the Nansen Basin (Fig. 1). A closer look at part of the Nansen Basin staircase (Fig. 5d) reveals layers separating or merging, depending on direction, and new layers forming over a distance of about 6 km (5 h) in between two CTD casts. This staircase lies in a strong double diffusive gradient (with warmer saltier water over cooler fresher water) above the core of an anticyclonic intra-thermocline eddy with a clear potential density signature (Fig. 6a). This signature extends from 150–500 m depth and spans roughly 200 km laterally. The core is relatively warm (~1.4 °C) and saline (~35), suggestive of Atlantic water origin (Fig. 6b,c). It is likely that the eddy was formed in an environment where a staircase was present, and that the eddy brought a section of the staircase within its core to where it was presently observed, although we cannot rule out the possibility that the staircase was actually formed within the eddy. This dynamical and thermohaline structure parallels that of Meddy Sharon^28^.

Along with the ability to continuously track thermohaline staircases over large spatial scales, the high horizontal and vertical resolution of our acoustic data also reveals possible evidence of shear instabilities (Fig. 4d) which, although poorly understood, are believed to be the main cause of internal wave dissipation and associated diapycnal mixing in the interior ocean^29^.

Shear-induced decay of internal waves has been observed acoustically in highly dynamic estuaries^16,30^ but no direct observations have previously been made of decaying internal waves in the open ocean. We do not see the large-scale billows seen in ref.^16^, but these are believed to be rare and shear instabilities in the open ocean are, according to *Barad and Fringer*, more likely to resemble the numerical simulations presented in ref.^29^ or perhaps ref.^31^. The visual similarities between such simulations and the data shown in Fig. 4d, characterized by a transition at the wave trough where much of the high frequency energy at the trailing side of the wave suddenly disappears, is striking. Furthermore, it has been shown that shear instability is not only a function of the critical Richardson number, but stability also decreases with decreasing thickness of the interface layer separating two homogenous layers^31^. Within a staircase with very thin interface layers between the steps, shear instabilities could thus occur under conditions that would be considered stable in coastal areas with typically thicker layer interfaces.

The manner in which thermohaline staircases are formed and their relative contribution to heat flow is also not well known^9,10^. Recent direct numerical simulations^32^ show that the vertical heat flux can be a factor of two larger than the established parameterized “4/3 flux law” based on laboratory experiments^7^. On the other hand, ref.^10^ found that the same parametrization overestimated the total heat flux by up to an order of magnitude compared to estimates based on microstructure observations, and at least part of this discrepancy is thought to be due to observational limitations. The high-resolution imaging described here will help to answer two central, yet unresolved questions: (1) how is staircase step thickness, which controls vertical fluxes^6^, influenced by lateral splitting/merging of layers; and (2) how do lateral thermohaline intrusions coexist and interact with thermohaline staircases? Reference^10^ found a consistent staircase in the Amundsen Basin, close to locations where, in this study, there were none. The apparent temporal variability of these staircase structures can be assessed with sonar mapping, complemented by CTD surveys and ice tethered profiler programs. Furthermore, this observational technique will allow us to address the critical question of the spatial and temporal statistics (intermittency) of mixing events.

To summarize, we have used co-located acoustic and CTD data to unequivocally demonstrate that there is a one-to-one correspondence of thermohaline stairsteps and acoustic layering. We were able to track a staircase continuously (with some interruptions due to ice breaking in between drift stations) for 36 h over a ship track of 91 km (Fig. 4a), and identify an isolated staircase, extending about 100 km laterally, in the Nansen Basin (Fig. 5). We also present observations of layers forming/disappearing and splitting/merging within a thermohaline staircase (Fig. 5d–f). Finally, we present, for the first time, continuous high frequency vertical displacements of individual steps within an Arctic thermohaline staircase caused by internal waves. It is hypothesized that a sudden transition within an internal wave, where much of the high frequency energy at the trailing side of the wave suddenly disappears, is related to shear instabilities.

The data presented here suggest that in order to fully understand vertical heat fluxes, not only the presence of staircases must be considered, but also the mixing caused by decaying internal waves, which is likely not accounted for in laboratory measurements, numerical simulations, or very localized (i.e., discrete profile) measurements. A synoptic view is needed where, ideally, acoustic observations similar to the ones presented here are augmented by concurrent CTD and microstructure observations. This acoustic observational technique should offer new insights into the mechanisms controlling layer formation and evolution, and eventually allow for greater understanding of interactions between double-diffusive convection and shear-induced turbulence, as well as their control on diapycnal fluxes.

**Figure 7:**
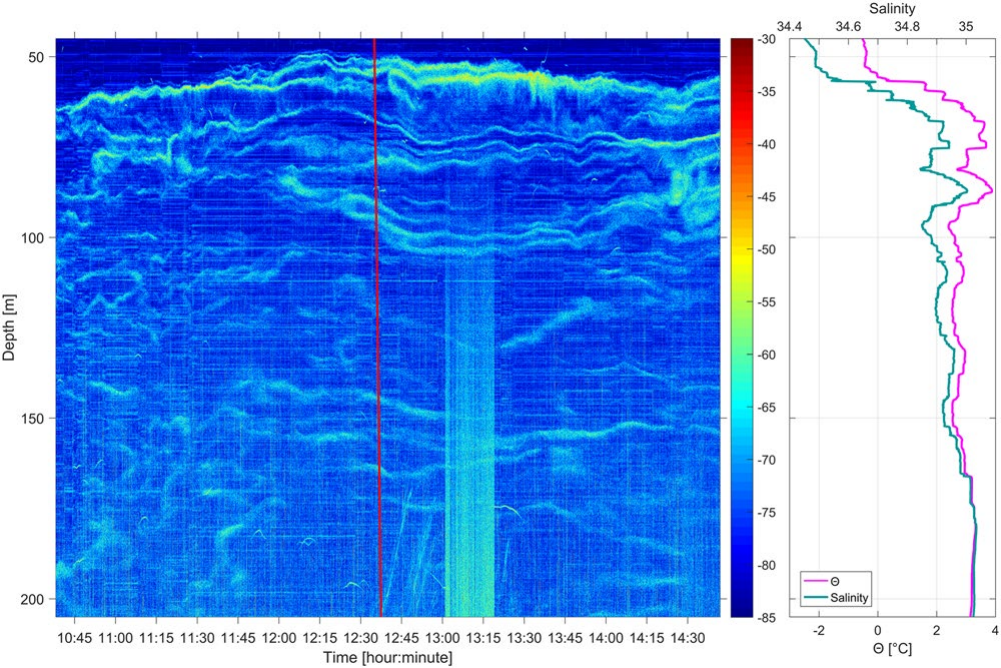
Acoustic observations of fine-scale thermohaline structure. (**a**) Processed EK80 echogram (1 ms pulse length) with CTD cast (red line). (**b**) CTD potential temperature and salinity profiles.

Furthermore, the observational methodology presented here can be used to track fine-scale thermohaline structures in general, as exemplified in Fig. 7. With the growing use of broadband echo sounders, high-resolution mapping of ocean stratification will become possible over wide spatial areas. This will allow for broad spatial and temporal reconstruction of salinity and temperature structure between *in-situ* CTD and ARGO float measurements, that are currently largely under-sampling ocean thermohaline variability^33^.
